# Supplementary material for: Lymphedema and Post-Operative Complications after Sentinel Lymph Node Biopsy versus Lymphadenectomy in Endometrial Carcinomas—A Systematic Review and Meta-Analysis
Source: J Clin Med. 2020 Dec 31;10(1):120. doi: 10.3390/jcm10010120 (PMC7795280; doi:10.3390/jcm10010120)
Supplement: Supplementary file 1 [file jcm-10-00120-s001.pdf]

Journal of
Clinical Medicine

MDPI

Supplement Post-operative complications grading systems

Table S1. Memorial Sloan Ketting Cancer Center Surgical Secondary Events Grading System, used by Accorsi et al. [24].

**Grade 0**	No complications
**Grade I**	Bedside care or oral medications
**Grade II**	Intravenous medications, transfusion
**Grade III**	Radiologic, endoscopic or operative intervention required
**Grade IV**	Chronic disability or organ resection
**Grade V**	Death

Table S2. Accordion Severity Classification, used by Casarin et al.]37,38].

**Grade 0**	No complications
**Grade I**	Complications carrying minor risks. At most requires bedside procedure. Allowed therapeutic regimens are: antiemetics, antipyretics, analgesics, diuretics, electrolytes, and physiotherapy. Hospital stay required for treatment of complication does not exceed twice the median length of stay for the procedure
**Grade II**	Potentially life threatening
**Grade IIa**	Requiring pharmacological treatment with drugs other than such allowed for grade 1 complications. Blood transfusions and total parenteral nutrition are also included. Any patient with a complication and hospital stay exceeding twice the median length of stay for the procedure and not falling into a higher category
**Grade IIb**	Requiring invasive procedures: surgical, endoscopic or radiological (invasive) intervention
**Grade III**	Complications with residual or lasting disability. Examples are stroke, organ and limb loss
**Grade IV**	Death in a patient with a complication

Table S3. Clavien Dindo Classification, used by Geppert *et al.*, Imboden et al. and Polan et al. [35,39–41].

**Grade 0**	No complications
**Grade I**	Any deviation from the normal post-operative course without the need for pharmacological treatment or surgical, endoscopic and radiological interventions. Allowed therapeutic regimens are: drugs as antiemetics, antipyretics, analgesics, diuretics and electrolytes and physiotherapy. This grade also includes wound infections opened at the bedside
**Grade II**	Requiring pharmacological treatment with drugs other than such allowed for grade I complications. Blood transfusions and total parenteral nutrition are also included
**Grade III**	Requiring surgical, endoscopic or radiological intervention
**Grade IIIa**	Intervention not under general anesthesia
**Grade IIIb**	Intervention under general anesthesia
**Grade IV**	Life-threatening complication (including CNS complications)* requiring IC/ICU-management
**Grade IVa**	Single organ dysfunction (including dialysis)
**Grade IVb**	Multiorgan dysfunction
**Grade V**	Death of a patient

*brain hemorrhage, ischemic stroke, subarachnoid bleeding, but excluding transient ischemic attacks; IC intermediate care; ICU intensive care unit.
